# Circumferential Surgical Management of a Cervical Chordoma: A Case Report and Review of the Literature

**DOI:** 10.7759/cureus.94628

**Published:** 2025-10-15

**Authors:** Kevin S Toache, Flavio Hernandez-Gonzalez, Edgar F Higuera-González, Carlos J Mávita Corral, Oswaldo Sánchez-Lezama, Isauro Lozano Guzmán, Tomas Moncada-Habib, Victor Correa-Correa

**Affiliations:** 1 Neurosurgery, Hospital de Especialidades, Centro Medico Nacional SXXI, Mexico City, MEX; 2 Neurosurgery, Instituto Nacional de Neurología y Neurocirugía Manuel Velasco Suárez, Mexico City, MEX

**Keywords:** chordoma, circumferential approach, spinal fusion, spinal neoplasms, spinal stabilization

## Abstract

Cervical chordomas are rare malignant tumors derived from notochordal remnants and represent a surgical challenge due to their locally aggressive behavior and proximity to vital neurovascular structures. We present the case of a 57-year-old man with progressive neurological decline over two months, characterized by weakness in all extremities and gait impairment. MRI demonstrated a lobulated extradural mass extending from C3 to C6 with vertebral body destruction and severe spinal cord compression. A staged circumferential surgical approach was performed. The first stage involved anterior corpectomy and tumor resection with reconstruction using a titanium cage and anterior plate, while the second stage consisted of posterior instrumentation for stabilization. Histopathological examination confirmed conventional chordoma with brachyury expression. Postoperatively, the patient showed marked neurological recovery, achieving independent ambulation at two months, and was referred for adjuvant radiotherapy.

This case illustrates the complexity of cervical chordoma management and emphasizes the importance of multidisciplinary strategies. Circumferential resection with reconstruction, combined with precision radiotherapy, offers an effective approach to improve functional outcomes and long-term disease control in this rare but formidable entity.

## Introduction

Chordomas are rare, slow-growing malignant tumors derived from the embryonic remnants of the notochord. They account for approximately 1-4% of all primary bone tumors, with an incidence of less than 0.1 per 100,000 persons per year [1,2]. Although chordomas most commonly involve the sacrococcygeal region (50%) and the clivus/skull base (30-35%), approximately 10-15% occur in the mobile spine, particularly in the cervical segment [3,4].

Cervical chordomas present unique diagnostic and therapeutic challenges because of their location adjacent to critical neurovascular structures, including the spinal cord, vertebral arteries, and cranial nerves in the upper cervical levels [5]. The clinical presentation is often nonspecific and includes neck pain, radiculopathy, myelopathy, and occasionally dysphagia or lower cranial nerve dysfunction, depending on the level of involvement [6]. Their indolent growth often leads to delayed diagnosis, and by the time they are detected, significant bony destruction and soft tissue extension may have occurred [7].

MRI and CT imaging are essential for diagnosis and surgical planning. MRI typically shows a lobulated, T2-hyperintense mass with contrast enhancement, whereas CT reveals lytic bone destruction with calcifications in some cases [8]. The definitive diagnosis is established by histopathological evaluation, typically revealing physaliphorous cells within a myxoid matrix that are positive for cytokeratin, epithelial membrane antigen (EMA), and brachyury [9].

The mainstay of treatment is surgical resection, aiming for maximal safe tumor removal, ideally en bloc, followed by high-dose adjuvant radiotherapy, particularly with proton beam or carbon-ion therapy, which offer superior conformality and tissue sparing [10]. However, owing to the complex regional anatomy and proximity to the spinal cord, nerve roots, and vertebral arteries, en bloc resection is rarely feasible in the cervical spine [11]. In selected cases with extensive tumor burden or biomechanical instability, a 360-degree surgical approach (circumferential surgery) involving anterior tumor debulking and reconstruction, followed by posterior instrumentation and resection of the residual tumor, is indicated to achieve both oncological control and spinal stability [8,12].

This report presents a case of cervical chordoma treated with a staged circumferential approach, followed by a review of the literature to contextualize the role of 360-degree surgery in achieving optimal outcomes for such challenging lesions.

## Case presentation

A 57-year-old man presented with a two-month history of progressive neurological decline, initially manifesting as distal weakness of the right hand in November 2024. The deficit gradually extended proximally in the right upper limb, followed by impaired fine motor skills and contralateral upper extremity weakness. By December, he developed gait instability, difficulty climbing stairs, and eventually required assistance for ambulation. One month later, he reported constipation, complete loss of standing ability, and further deterioration of upper limb function.

Neurological examination revealed spasticity of the lower limbs (Ashworth grade 3), hypotonia on the left side, generalized muscle atrophy, and fasciculations in the thighs and right arm. Muscle strength, assessed using the Daniels scale, was 1/1/1 in the right upper extremity, 1/1/0 in the left upper extremity, and 1/0/0 in both lower limbs (proximal/intermediate/distal, respectively). Deep tendon reflexes were brisk, with bilateral Hoffmann and extensor plantar responses, and a positive Tromner sign on the right. Sensory modalities were preserved. These findings were consistent with high cervical myelopathy and marked upper motor neuron involvement.

**Figure 1:**
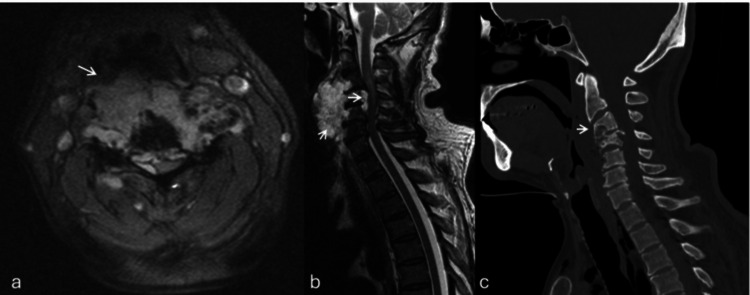
Preoperative imaging of a cervical chordoma with extensive bone and soft tissue involvement. (a) Axial T2-weighted fat-suppressed MRI (STIR sequence) reveals a heterogeneously hyperintense mass involving the cervical vertebrae with marked infiltration of the prevertebral and paravertebral soft tissues (arrow). (b) Sagittal T2-weighted MRI shows destruction of the mid-cervical vertebral bodies and significant spinal cord compression (arrows). (c) Sagittal CT in the bone window demonstrates lytic vertebral body destruction with collapse and structural compromise of the cervical spine (arrow). MRI: magnetic resonance imaging, STIR: short tau inversion recovery, CT: computed tomography

MRI of the cervical spine demonstrated a lobulated, expansile extradural mass extending from C3 to C6 with vertebral body destruction, paravertebral soft-tissue invasion, and severe spinal cord compression, findings compatible with chordoma (Figure 1).

**Figure 2:**
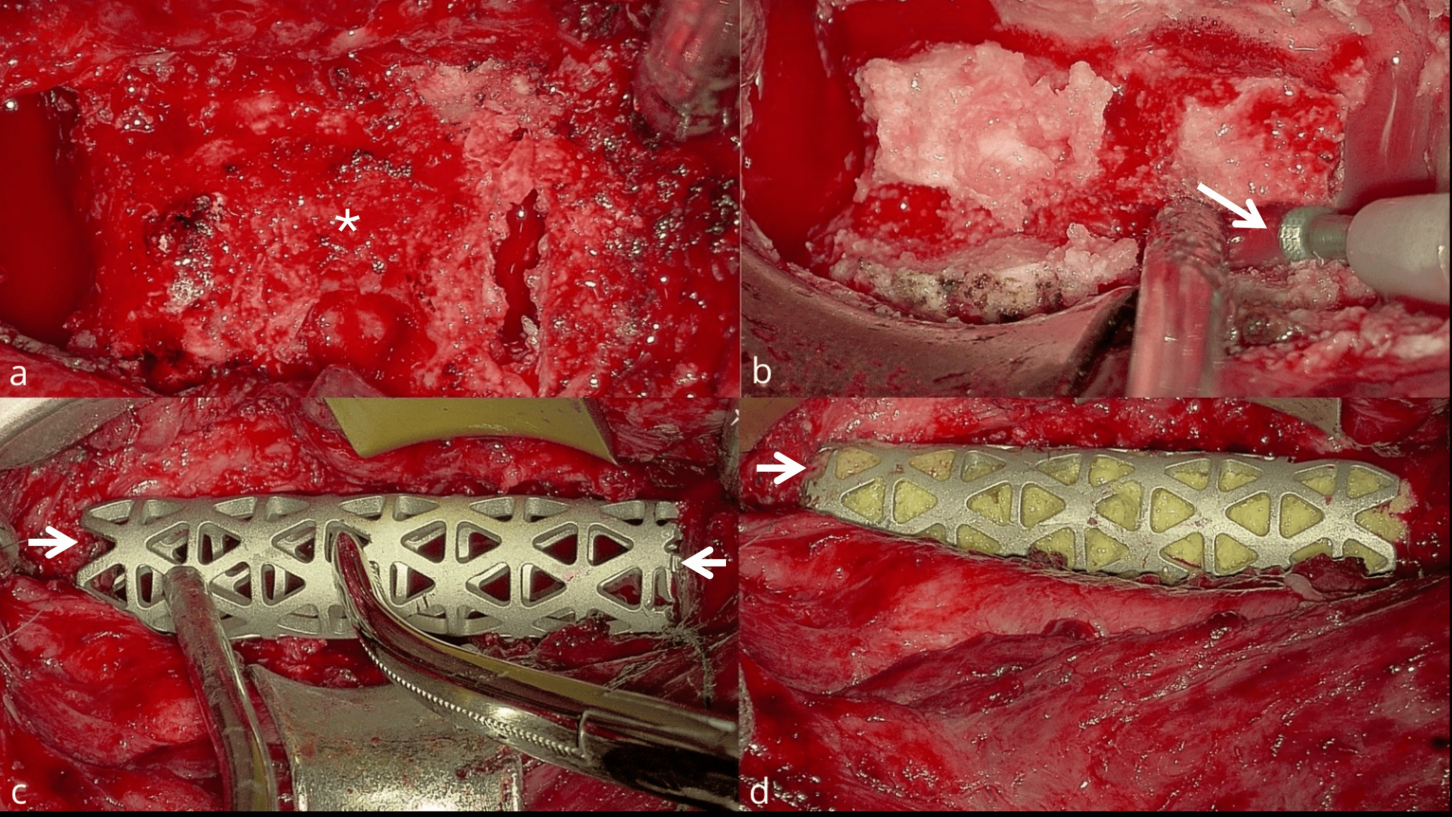
Intraoperative images. (a) Infiltrated tumor in the cervical vertebral bodies after discectomy of the upper and inferior levels. Fusion of C4 and C5 is observed, and a highly vascularized, infiltrating lesion involving the vertebral bodies is shown (star). (b) Corpectomy performed using an ultrasonic aspirator (arrow). (c) Measurement of the titanium cage after preparation of the superior and inferior vertebral body platforms (arrows). (d) Final position of the cage with bone allograft (arrow).

A staged circumferential surgical approach was performed. The first stage included anterior cervical corpectomy from C3 to C6 with intralesional (piecemeal) tumor resection, followed by reconstruction with a titanium mesh cage and anterior cervical plate spanning C2-C7 (Figure 2).

**Figure 3:**
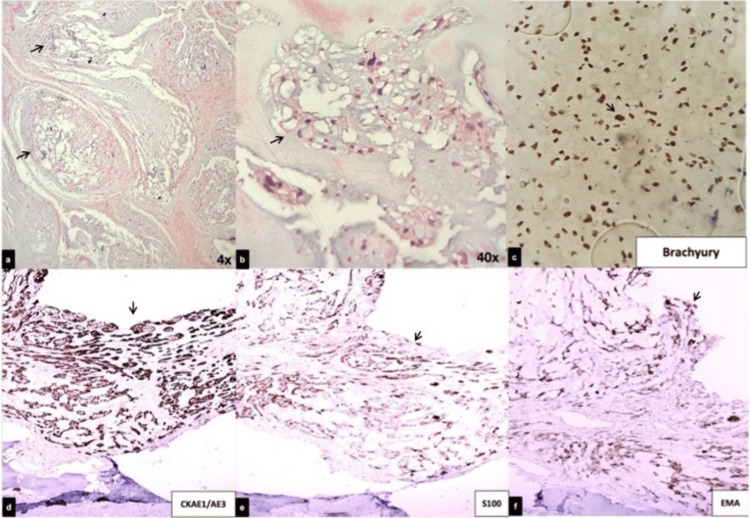
Histopathological and immunohistochemical profiles. (a–b) Hematoxylin and eosin staining shows a lobulated neoplasm composed of physaliphorous cells embedded in a myxoid stroma (a: low magnification, 4×; b: high magnification, 40×). (c) Immunohistochemistry reveals diffuse nuclear positivity for brachyury, confirming notochordal origin. (d–f) Additional staining shows cytoplasmic expression of cytokeratin AE1/AE3 (d), nuclear and cytoplasmic positivity for S100 (e), and membranous staining for EMA (f).

Intraoperatively, C4 and C5 were fused and replaced by a soft, gray, highly vascular extradural tumor. Histopathological examination confirmed a conventional chordoma (Figure 3).

The second stage involved posterior stabilization with C2 pedicle screws, C4 and C6 lateral mass screws, bilateral rods, and a cross-link. Postoperative imaging demonstrated adequate decompression and stable fixation of the cervical spine.

**Figure 4:**
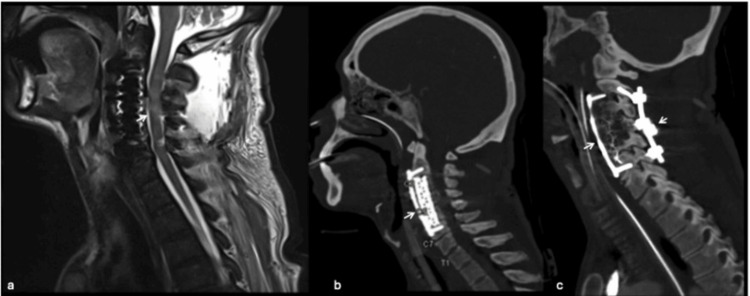
Postoperative imaging of a cervical chordoma following circumferential resection and reconstruction. (a) Sagittal T2-weighted MRI demonstrating adequate spinal cord decompression after tumor removal (arrow). (b) Sagittal CT scan showing anterior corpectomy with titanium mesh cage and anterior plate fixation extending from C2 to C7 (arrow). (c) Sagittal CT scan depicting posterior instrumentation with pedicle and lateral mass screws, bilateral rods, and cross-link, providing circumferential stabilization of the cervical spine (arrows).

At the two-month follow-up, the patient exhibited marked neurological recovery. Muscle strength improved from 1/5 to 5/5 in all extremities, allowing independent ambulation without assistance. Functional status returned to baseline, with full reintegration into activities of daily living. Postoperative MRI confirmed complete tumor resection and adequate spinal cord decompression, with no radiological evidence of residual disease or recurrence (Figure 4).

No postoperative complications were recorded. Adjuvant radiotherapy was initiated four months after surgery, consisting of 54 Gy delivered in 30 fractions. The patient remains under multidisciplinary follow-up, including neurosurgery and radiation oncology, with surveillance MRI scheduled every six months.

## Discussion

Chordomas of the cervical spine represent a rare and surgically demanding subset of primary bone tumors. These neoplasms arise from notochordal remnants and typically exhibit indolent growth with high local aggressiveness [1,2]. The clinical behavior and management strategies for cervical chordomas are significantly influenced by their unique anatomical context, including proximity to the spinal cord, vertebral arteries, and upper airway structures [3,4]. Histopathological confirmation remains critical, with brachyury expression now recognized as a highly sensitive and specific marker for chordomas, helping to distinguish them from chondrosarcomas and other spinal neoplasms [9]. The presence of physaliphorous cells within a myxoid matrix is a classic histological hallmark of this tumor [7].

This case illustrates the complexity of surgical decision-making in cervical chordomas. The use of a 360-degree (circumferential) approach, incorporating both anterior and posterior surgical corridors, allowed effective decompression, tumor resection, and spinal stabilization. This strategy has been increasingly adopted in cases where the lesion involves both the anterior and posterior vertebral elements or when gross total resection requires multilevel access [8,12].

En bloc resection remains the gold standard for treating chordomas, given their aggressive local behavior and limited response to chemotherapy and conventional radiotherapy. However, performing en bloc resection in the cervical spine poses significant technical challenges because of the lesion’s proximity to critical neurovascular structures and the spinal cord [1,6,11]. Currently, no guidelines exist for selecting the optimal instrumentation for cervical chordomas. Most documented cases describe anterior approaches with fixation [13], whereas posterior stabilization is less frequently highlighted. Nonetheless, data from other pathologies, such as metastatic disease and vertebral body infections, support the rationale for using combined anterior and posterior constructs to enhance stability and fusion rates [14-16]. High complication rates have been reported in multilevel cervical surgeries involving corpectomy and anterior fixation. In contrast, circumferential (360-degree) instrumentation has shown improved biomechanical outcomes and higher fusion rates, nearing 100% in reported series of complex cervical reconstructions [17-19]. In the present case, posterior instrumentation was added to anterior corpectomy and cage placement because of the multilevel extent of the disease [14,18], preoperative kyphotic deformity [14,20], and the inherent risk of recurrence associated with the natural history of this pathology. Although expandable cages have been successfully used for anterior column reconstruction [16], a mesh cage was chosen in this case to restore physiological cervical lordosis and ensure immediate biomechanical stability. A comparative summary of previously reported cases and literature addressing circumferential and en bloc surgical approaches for cervical chordomas is presented in Table 1.

**Table 1:** Comparative review: circumferential surgical management of cervical chordoma.

Study/Authors	Year	Approach Description	Key Outcomes and Survival Rates	Major Complications
Korwutthikulrangsri et al. [21]	2023	Two-stage, anterior-posterior, C2 total spondylectomy with transoral mandibular split and mesh cage reconstruction, combined with radiotherapy.	Midterm (5-year) outcome for a single patient: no tumor recurrence identified on MRI.	High morbidity of the combined approach; increased bleeding and operative time.
Pinter et al. [22]	2022	En bloc resection of a high cervical chordoma (C1-C3) followed by reconstruction with a free vascularized fibular graft.	Negative margins reported; long-term oncologic outcomes remain limited	Hardware failures and anterior column reconstruction-related complications
Joaquim et al. [23]	2021	Narrative review on circumferential cervical fusion.	Circumferential cervical fusion can be advantageous in some instances, such as high-risk patients for pseudoarthrosis or those with cervical deformity.	Higher morbidity, blood loss, and longer operative time in combined approaches
Aoun et al. [24]	2018	Four-level en bloc (C3–C6) 360°.	Four-level en bloc (C3–C6) 360°.	High structural failure rate; need for reoperations
Wang et al. [25]	2017	Two-stage en bloc excision of multilevel cervical chordomas via parasagittal osteotomy with expandable cage reconstruction and posterior instrumentation in 4 patients	All 4 patients underwent en bloc excision of chordoma.	Nerve root sacrifice in 2 patients; vertebral artery ligation in 3 patients
Cloyd et al. [26]	2009	Two cases of cervical chordoma managed via en bloc resection (C3-C6 and C2), with a systematic review.	Survival data and rates of recurrence after en bloc resection for cervical spinal tumors are limited to single case reports and small case series, making the true risk of recurrence unknown.	Limited data from case reports and small series make comprehensive complication analysis challenging for the en bloc resection of cervical spinal tumors. The excerpt does not list specific major complications.

These studies highlight the technical diversity and clinical outcomes of complex reconstructive strategies involving both anterior and posterior corridors. The compilation underscores how circumferential (360-degree) surgery has progressively evolved as a viable option for achieving decompression, stabilization, and local control in selected cases, despite its inherent surgical morbidity.

Despite surgical resection, the recurrence rate remains high. McMaster et al. reported a five-year survival rate of 51% for chordomas, with the majority of recurrences occurring locally [1]. Bergh et al. highlighted that the extent of resection was the most significant predictor of local recurrence and survival in mobile spine chordomas [3]. Given these recurrence patterns, adjuvant radiotherapy is routinely recommended for these patients. High-dose radiotherapy using proton-beam or carbon-ion therapy has shown superior local control compared with conventional photon-based techniques. Carbon-ion therapy offers biological advantages in radioresistant tumors such as chordomas and provides steep dose gradients suitable for lesions near the spinal cord [10]. However, complications such as dysphagia, wound issues, and instrumentation failure remain challenging, particularly in multilevel surgeries [6,8]. Long-term follow-up is essential, as late recurrences have been reported even beyond five years after treatment [4,5].

## Conclusions

Cervical chordomas represent a rare but formidable surgical challenge due to their locally aggressive nature, proximity to critical neurovascular structures, and high risk of recurrence. Circumferential (360-degree) approaches allow safer and more effective resections in selected patients, offering both oncologic and biomechanical advantages, though at the cost of increased technical complexity and perioperative risk.

The integration of advanced radiotherapy techniques, such as proton or carbon-ion therapy, remains essential to optimize local control in this radioresistant pathology. Long-term surveillance and continued clinical reporting are indispensable to refining treatment strategies and improving outcomes. These cases underscore the need for individualized, multidisciplinary management to maximize both survival and functional preservation.
